# The Utility of Computed Tomography of the Chest at the Time of Induction Chemotherapy in Patients with Acute Leukemia

**DOI:** 10.3390/cancers18193155

**Published:** 2026-09-29

**Authors:** Maro Ohanian, Fieke W. Hoff, Vikas Kundra, Dimitrios P. Kontoyiannis, Graciela N. Nogueras-Gonzalez, Javier Adachi, Dawid Schellingerhout, Samuel Shelburne, Sherry Pierce, Saadia A. Faiz, Yesid Alvarado, Tapan Kadia, Koji Sasaki

**Affiliations:** 1 Department of Leukemia, University of Texas MD Anderson Cancer Center, Houston, TX 77030, USAyalvarad@mdanderson.org (Y.A.);; 2 Department of Radiology and Nuclear Medicine, University of Maryland School of Medicine, Baltimore, MD 21201, USA; 3 Department of Infectious Diseases, University of Texas MD Anderson Cancer Center, Houston, TX 77030, USAsshelburne@mdanderson.org (S.S.); 4 Department of Biostatistics, University of Texas MD Anderson Cancer Center, Houston, TX 77030, USA; 5 Department of Neuroradiology and Imaging Physics, The University of Texas MD Anderson Cancer Center, Houston, TX 77030, USA; 6 Department of Pulmonary Medicine, University of Texas MD Anderson Cancer Center, Houston, TX 77030, USA; safaiz@mdanderson.org; 7 Department of Laboratory Medicine, Graduate School of Medical and Dental Sciences, Institute of Science Tokyo, Tokyo 113-8549, Japan

**Keywords:** computed tomography, chest CT, chest X-ray, leukemia, acute leukemia, induction chemotherapy, radiographic

## Abstract

**Background**: Compared to chest X-ray, computed tomography (CT) imaging of the chest is more sensitive and specific for identifying pulmonary abnormalities. However, patients with acute leukemia routinely undergo chest X-ray before beginning chemotherapy, and the benefit of obtaining baseline chest CT remains unclear. **Methods**: In this retrospective study, 50 patients with newly diagnosed acute leukemia who underwent chest X-ray and chest CT without contrast before beginning induction chemotherapy were included. CT imaging reports were independently evaluated by two hematologist–oncologists for infection concern and actionability, and these assessments were compared to the actions taken by the treating physicians. **Results**: Regardless of the presence of chest X-ray abnormalities, chest CT identified abnormalities in 94% of patients (47/50), a significantly higher detection rate than that for chest X-ray, which demonstrated abnormalities in only 66% of patients (33/50) (*p* < 0.001). Of the 17 patients with normal chest X-ray findings, 14 (82%) had abnormal chest CT findings. Of these 14 patients, 10 had actionable abnormalities, and four had non-clinically significant findings. All 33 patients with abnormal chest X-ray findings also had abnormal chest CT findings, and three patients with normal chest X-ray findings also had normal chest CT findings. Of the 30 patients with normal or minimal chest X-ray findings compared to chest CT findings, 26 patients (87%) had chest CT findings that were actionable, prompting more aggressive care and/or closer monitoring. On multivariate analysis, patients with both abnormal chest X-ray and chest CT findings had worse overall survival than patients without both abnormal chest X-ray and chest CT findings (*p* = 0.005). **Conclusions**: These findings highlight a diagnostic gap, potentially supporting prospective studies on routine chest CT imaging for patients with newly diagnosed acute leukemia.

## Simple Summary

Performing a chest X-ray before starting chemotherapy is standard practice for patients with acute leukemia, whereas computed tomography of the chest (chest CT) is not. In this study, we compared patients’ chest CT and chest X-ray findings obtained at the time of acute leukemia diagnosis. Chest X-ray findings were normal or minimal compared to chest CT findings in 30 of 50 cases. In 26 of these 30 cases (87%), chest CT findings resulted in more aggressive clinical management. Of the 14 patients with normal chest X-ray but abnormal chest CT findings, 10 had actionable chest CT findings. Chest CT abnormalities further stratified patients’ survival differences according to whether baseline chest X-ray findings were normal or abnormal; patients with abnormal chest X-ray and chest CT findings had the worst survival. Our results highlight a diagnostic gap, potentially supporting prospective studies on routine chest CT imaging for patients with newly diagnosed acute leukemia.

## 1. Introduction

Patients with acute leukemia are at increased risk for pulmonary infections, which are the leading cause of morbidity and death for these patients [1,2,3,4,5]. Moreover, patients with acute leukemia may present with non-infectious, acute respiratory pathology, such as leukocytosis, potentially leading to diffuse alveolar hemorrhage or acute respiratory distress syndrome during induction chemotherapy [6,7]. In asymptomatic patients with acute leukemia, imaging is typically limited to chest X-rays [8,9,10]. Likewise, initial imaging performed in symptomatic patients is also usually limited to a chest X-ray; however, smaller effusions, ground-glass infiltrates or other occult thoracic abnormalities may be missed.

Compared to chest X-ray, computed tomography of the chest (chest CT) is well established to be more sensitive and specific for detecting pulmonary abnormalities [11,12]. However, although routine for patients with solid organ tumors, baseline chest CT imaging is not considered standard of care for patients with acute leukemia at the time of diagnosis or before the initiation of induction chemotherapy [13,14]. Because patients with acute leukemia are immunosuppressed [15], diminished immune and inflammatory responses can delay or obscure chest X-ray findings, making CT imaging preferable [13,14]. Thus, the potential benefit of an initial chest CT requires further clarification in acute leukemia care.

Chest CT is often not ordered until clinical symptoms develop or when chest X-ray findings are equivocal. While empiric broad-spectrum antibiotics are standard of care for patients with acute leukemia who have a suspected infection, chest CT provides earlier and more definitive detection of respiratory disease compared to chest X-ray, potentially enabling earlier interventions and preventing severe infectious complications. This could be applicable to immunosuppressed patients susceptible to opportunistic pathogens.

While it is known that chest CT is diagnostically superior to chest X-ray, an unresolved question in acute leukemia management is the clinical utility of chest CT. Our objective was to evaluate and compare chest X-ray and chest CT findings of patients with newly diagnosed acute leukemia who underwent such imaging within four days before beginning induction chemotherapy and to assess the clinical utility of chest CT. We hypothesized that CT abnormalities detected at the initiation of induction chemotherapy would identify clinically actionable findings not detected on chest X-ray. We also explored potential associations between imaging findings and overall survival (OS).

## 2. Methods

We conducted a retrospective analysis of clinical data and chest imaging reports of patients with newly diagnosed acute myeloid leukemia (AML) or acute lymphoblastic leukemia (ALL). We included patients in the electronic medical record who presented to The University of Texas MD Anderson Cancer Center between 25 March 2016 and 1 June 2020 with newly diagnosed acute leukemia and who underwent both chest X-ray and chest CT without contrast within four days before the initiation of induction chemotherapy/targeted therapy. The study was approved by the Institutional Review Board at MD Anderson Cancer Center (protocol 2020-0782) in accordance with the Declaration of Helsinki.

Clinical characteristics were collected, including basic demographics; vital signs at the time of evaluation (including oxygen saturation by pulse oximetry, supplemental oxygen requirements, and temperature); presence or absence of fever or cough within one week of imaging; and laboratory data (hematologic and microbiological parameters). The treating physicians’ reasons for ordering chest CT were collected. Official radiology reports for chest X-rays and chest CTs were independently assessed and categorized as normal and abnormal by two board-certified hematologist–oncologists with expertise in leukemia who were blinded to the patients’ clinical condition.

Chest CT findings were further evaluated for clinical actionability. When evaluating chest CT actionability and whether chest CT reports were concerning for infection, reviewers were also blinded to chest X-ray findings. If there were any disagreements between the two reviewers, then a third reviewer (a board-certified hematologist–oncologist with leukemia expertise) was consulted. Based on the independent assessment by the two hematologist–oncologists, chest CT findings were determined to be actionable if they met the pre-defined criteria of resulting in either a major or minor change in clinical management. Such major changes were specialty consultation (e.g., consultation with an infectious disease physician); initiation, escalation, or modification of antimicrobial therapy; and performance of a diagnostic or therapeutic procedure. Such minor changes were increased clinical surveillance or monitoring. Chest X-ray findings were considered minimal relative to chest CT findings when the chest X-ray findings were normal or indeterminate or otherwise demonstrated substantially less extensive disease than that identified by chest CT. Disease was considered substantially more extensive on chest CT than on chest X-ray when chest CT findings demonstrated additional pulmonary abnormalities, greater anatomic extent of disease, multifocal involvement, diffuse infiltrates, nodules below radiographic resolution, pleural or mediastinal abnormalities, or other clinically significant findings that were not identified or were substantially underestimated on chest X-ray findings.

### Statistics

Descriptive statistics and frequency tables were used to summarize the study variables overall and by type of diagnosis. To determine if the chest X-ray and chest CT radiographic findings were in agreement, both the McNemar chi-squared test and the kappa statistic were used [16]. To assess interobserver agreement among hematologist–oncologists regarding whether chest CT findings were concerning for infection and/or actionable, the kappa statistic was used [16]. The Kaplan–Meier product limit method [17] was used to estimate OS. OS was calculated from the date of diagnosis to the date of last follow-up or death. Cox proportional hazards regression was used to identify associations between the variables and OS. Multivariable models were defined using clinically relevant variables and risk factors associated with acute leukemia. Statistical analysis was performed using Stata/SE version 16.0 statistical software (StataCorp LLC, College Station, TX, USA). GraphPad Prism version 10.6.0 (GraphPad Software, San Diego, CA, USA) was used for preparation of pie charts. Chi-squared test or Fisher exact tests were used to calculate *p*-values. *p* < 0.05 was considered statistically significant.

## 3. Results

Of the 1190 patients with newly diagnosed acute leukemia who presented to our institution during our inclusion period, 50 patients (4.2%) met our inclusion criteria (i.e., undergoing both chest X-ray and chest CT without contrast within four days of beginning induction chemotherapy/targeted therapy). Of these 50 patients, 39 had AML and 11 had ALL. Demographics and clinical characteristics are shown in Table 1. The median age of the cohort was 68 years (range, 18–94 years), and over half of the patients (56%) were male. No patient had documented past medical history of viral, bacterial, or fungal respiratory/sinus infections 30–90 days prior to acute leukemia diagnosis. Patients received a variety of leukemia-directed treatment regimens, which are listed in Supplemental Table S1.

### 3.1. Chest Imaging Abnormalities

Chest CT findings were abnormal in 94% of patients (*n* = 47), regardless of whether chest X-ray findings were normal or abnormal (Table 2A). Overall, chest CT detected a significantly higher proportion of patients with abnormalities than did chest X-ray (33/50; 66%, *p* < 0.001). Compared to chest X-ray, chest CT identified marginally more pleural or pericardial effusions (*n* = 22; 44% vs. *n* = 13; 26%, *p* = 0.059) and identified significantly more nodules (38% vs. 12%, *p* = 0.003). No statistically significant differences were observed between chest X-ray and chest CT findings in terms of opacities (*p* = 0.220) or cavitations (*p* = 0.564).

Chest CT was performed for the following reasons: to evaluate abnormal chest X-ray findings and worrisome clinical symptoms (*n* = 33); to evaluate clinical signs or symptoms suggestive of infection despite normal chest X-ray (*n* = 15), e.g., cough alone (*n* = 3) or fever with or without cough/dyspnea (*n* = 12); or to establish a baseline before chemotherapy for patients with normal chest X-ray findings (*n* = 2) (Table 2B). Chest X-ray preceded chest CT in all but two patients. The first patient underwent chest X-ray 15 h after chest CT, and both modalities demonstrated no actionable findings. The second patient underwent chest X-ray 16 h after chest CT to confirm adequate central venous catheter placement; their chest X-ray was normal, whereas their chest CT had actionable findings concerning for infection.

Imaging findings of the 33 patients with abnormal chest X-ray findings and the 47 patients with abnormal chest CT findings are indicated in Supplemental Figure S1A and Supplemental Figure S1B, respectively. Seven patients had mediastinal lymphadenopathy, and two had septal thickening. In total, 19 nodules were observed (prominent nodules only, *n* = 5; indeterminate/subcentimeter nodules only, *n* = 11; both prominent nodules and small indeterminate nodules, *n* = 3).

### 3.2. Agreement Between Chest X-Ray and Chest CT Findings

Chest CT revealed multiple abnormalities not visible on chest X-ray (Supplemental Figure S1C). Of the 17 patients with normal chest X-ray findings, 14 (82%) had abnormal chest CT findings. Of these 14 patients, four had non-specific, non-clinically significant chest CT findings. Thirty-three (66%) patients had both abnormal chest X-ray and chest CT findings, and only three patients had both normal chest X-ray and chest CT findings.

**Figure 1:**
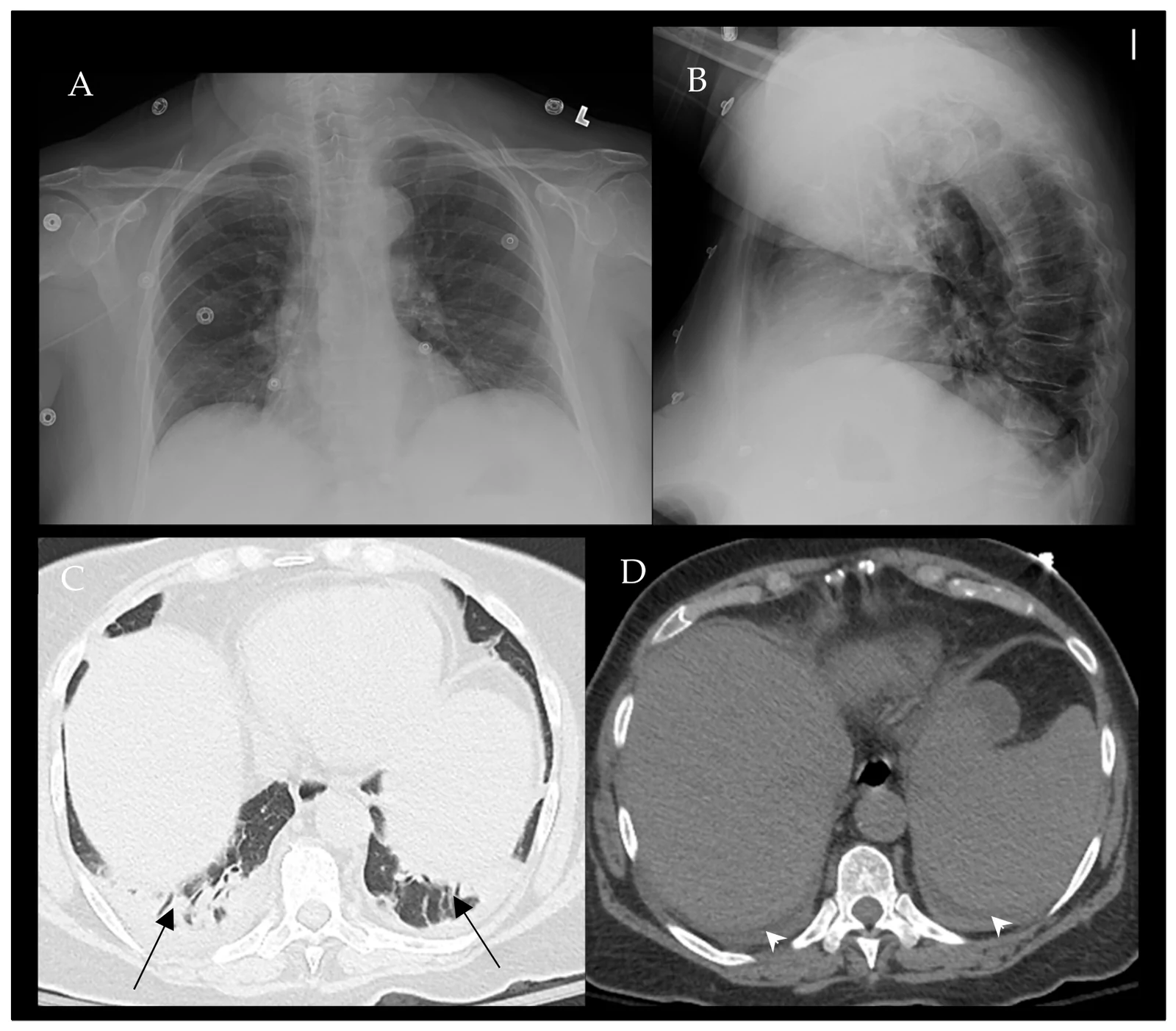
Frontal (**A**) and lateral (**B**) chest radiograph without evidence of infection or pleural effusion. Axial CT images of the lower thorax demonstrate bilateral opacities (arrows (**C**)) and pleural effusions (arrowheads (**D**)).

We evaluated the agreement between chest X-ray and chest CT findings for the presence or absence of opacities, effusions, nodules, and cavitations, as shown in Table 3. The agreement between the two modalities was 60% (Kappa *p*-value = 0.096; McNemar’s *p*-value = 0.180) for opacities, 94% (Kappa, *p*-value = 0.582; McNemar’s *p*-value = 0.564) for cavitations, 66% (Kappa *p*-value = 0.017; McNemar’s *p*-value = 0.029) for pleural and/or pericardial effusions, and 70% (Kappa *p*-value = 0.007; McNemar’s *p*-value = 0.001) for nodules. Of the 22 patients (44%) with either pleural and/or pericardial effusions (pleural effusion, *n* = 20; pericardial effusion, *n* = 1; both pleural and pericardial effusions, *n* = 1) on chest CT, 13 (59%) did not have pleural effusions on chest X-ray, and nine (41%) had pleural effusions on both chest CT and chest X-ray. An unexpected finding was that four of the 50 patients (8%) had pleural effusions reported on chest X-ray but not on chest CT. Figure 1 shows frontal (A) and lateral (B) chest radiographs with no evidence of infection or pleural effusion. In contrast, chest CT demonstrated prominent bilateral opacities (arrows, C) and pleural effusions (arrowheads, D).

### 3.3. Fever/Infection

Although 19 patients had a fever at baseline, 31 (62%) patients had a fever within a week of undergoing imaging. There was no statistically significant difference in the presence or absence of clinical symptoms (i.e., fever within one week, needing supplemental oxygen, or cough) with respect to imaging abnormalities (Supplemental Table S2).

Among the 47 patients with abnormal chest CT findings (including 33 who had both abnormal chest X-ray and chest CT findings and 14 who had only abnormal chest CT findings), 13 (28%) had a cough, 22 (47%) required oxygen, 28 (60%) had a fever, and eight had both a fever and a cough; five patients had fever and a cough and needed oxygen. Of the 19 patients without a fever but who had abnormal chest CT findings, only five had a cough, and of the 37 (79%) patients with chest CT findings suggestive of infection, 16 (43%) had a fever.

All three patients with both normal chest X-ray and chest CT findings had a fever within one week of imaging, suggesting fevers related to infection or leukemia. Of the 10 patients who had radiographic lesions on chest CT that were not suggestive of an active pulmonary infection, only three had a fever, and their radiographic findings included atelectasis (*n* = 1), prominent nodule (*n* = 1), effusion and atelectasis (*n* = 1), tiny nodules (*n* = 5), and granuloma (*n* = 2).

### 3.4. Bronchoscopy

Because of concern for infection based on chest CT findings, 14 (28%) patients underwent bronchoscopy with bronchoalveolar lavage (BAL). The radiographic and clinical features of these fourteen patients are described in Supplemental Table S3. Of these patients, four (29%) had confirmed infection, including methicillin-resistant *Staphylococcus aureus* (MRSA), *Pseudomonas*, and *Ralstonia* sp. (*n* = 1), *Mycobacterium intracellulare* (*n* = 1), *Pseudomonas* (*n* = 1), and *Rhinovirus*/*enterovirus* (*n* = 1). Additionally, four of these patients had suspected fungal lung infections, despite all four having negative fungal cultures and negative aspergillus antigen on BAL and blood testing.

### 3.5. Clinician Agreement on CT Reports That Were Considered Concerning for Infection and/or Actionable

In 49 of the 50 cases, agreement on concern for infection was 98% (expected agreement = 62.48%) (Kappa = 0.9467; *p*-value < 0.001) and 98% (expected agreement = 62.48%) for actionability (Kappa = 0.9467; *p*-value < 0.001) (Supplemental Table S4). In the one case with disagreement between the clinicians, the third hematologist–oncologist agreed with the first clinician, yielding strong agreement for infection (Kappa = 0.9316, *p* < 0.001) and actionability (Kappa = 0.9316, *p* < 0.001).

### 3.6. Impact of Abnormal CT Imaging on Clinical Care

Of the 50 included patients, 30 had normal or minimal chest X-ray findings compared to CT findings. Of these 30 patients, 26 patients had chest CT findings that were actionable, prompting more aggressive care and/or closer monitoring, and the remaining 4 patients had both normal chest X-ray findings and abnormal, albeit non-actionable, chest CT findings. Only one of the 26 patients with actionable chest CT findings had isolated minor ground-glass opacities that were not apparent on chest X-ray and experienced solely a change in monitoring/surveillance rather than an objective change in care. Patients who had a change in care underwent antibiotic escalation or modification (*n* = 25), active surveillance activities (*n* = 8), and a planned procedure (*n* = 1), as well as had consultation requests (*n* = 9). These actions demonstrate that these chest CT abnormalities had either greater diagnostic certainty (e.g., consolidation, multifocal pneumonia), greater extent of disease (e.g., bilateral or multilobar involvement), associated complications (e.g., pleural or pericardial effusions, adenopathy), or potentially serious alternative diagnoses (e.g., invasive fungal infection) that were considered more clinically significant and actionable than isolated minor ground-glass opacities alone.

Of 50 included patients, 14 (28%) had normal chest X-ray findings and abnormal chest CT findings. Of these 14 patients, 10 had chest CT findings that were considered actionable (Table 4), and four had nonspecific, non-actionable chest CT findings, including tiny or indeterminate nodules (*n* = 2), a calcified granuloma (*n* = 1), or nonspecific enlarged axillary lymph nodes with atelectasis (*n* = 1), that were not concerning for infection. Among the 10 patients with actionable chest CT findings, management changes were modifications to the antibiotic regimen (*n* = 9), active surveillance/monitoring (e.g., surveillance blood cultures, respiratory PCR and blood serology; *n* = 5), or an infectious disease consult (*n* = 1). Some patients had more than one intervention. Of the additional 16 (32%) patients who had minimal chest X-ray findings and abnormal chest CT findings; all had chest CT findings that were actionable and concerning for infection. Specifically, these findings led to the initiation of more aggressive antibiotic therapy (*n* = 16), infectious disease consultation (*n* = 8), consideration of bronchoscopy (*n* = 1), or closer monitoring/active surveillance (*n* = 2). In accordance with NCCN guidelines and institutional practice, all 50 patients received antibacterial, antifungal, and antiviral prophylaxis beginning at diagnosis or before initiating induction therapy. Antimicrobial prophylaxis was continued throughout both induction and consolidation treatment cycles [18]. Among the 25 patients who underwent antibiotic escalation or modification, the median duration of antibiotic treatment was nine days (range, 4 to 72 days).

When examining the two patients who had undergone chest CT solely to obtain a baseline chest CT, one had a calcified granuloma, and the other had tiny non-specific sub-centimeter nodules; neither result was concerning for infection or considered actionable.

In 17 patients with both abnormal chest X-ray and abnormal chest CT findings, chest CT findings did not lead to the additional escalation of care because decisions had already been made based on the X-ray findings. Three patients had both normal chest X-ray and chest CT findings.

### 3.7. Abnormal Baseline Chest Imaging Studies and Laboratory Characteristics

The comparison of clinical laboratory characteristics across three groups (patients with both positive X-ray and chest CT findings, patients with only positive chest CT findings, and patients with both normal X-ray and chest CT findings imaging) revealed that, among the clinical laboratory values, only lactate dehydrogenase was significantly associated (*p* = 0.021) with the imaging findings (Supplemental Table S5).

### 3.8. Abnormal Baseline Chest Imaging Studies Are Associated with Worse Survival

**Figure 2:**
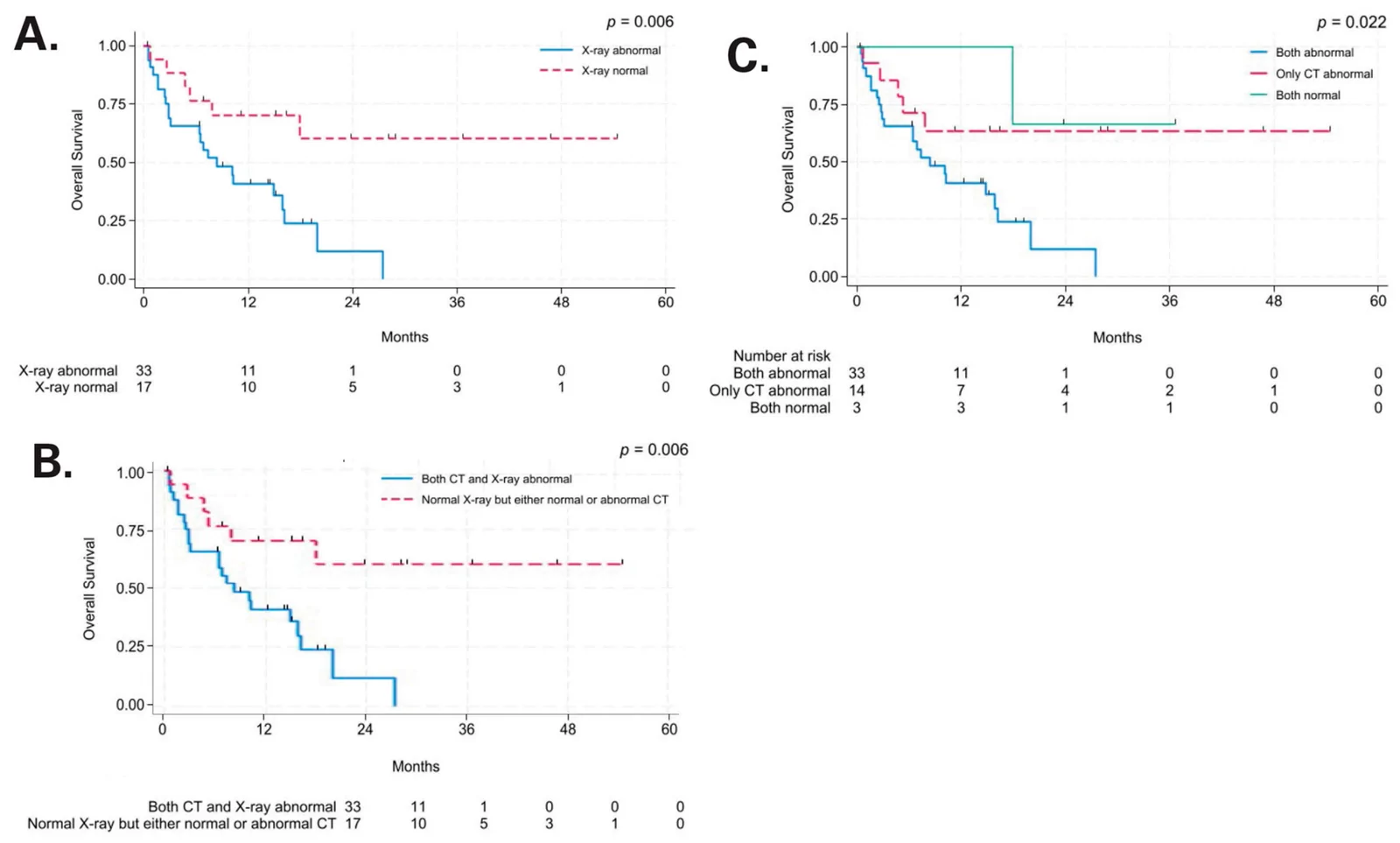
Overall Survival by Abnormal Chest CT and Abnormal chest X-ray. (**A**) Overall survival (OS) of patients with newly diagnosed acute leukemia according to abnormal vs. normal baseline chest X-ray results. (**B**) The patients without both abnormal chest X-ray and chest CT findings consisted of 3 patients with both normal chest X-ray and chest CT findings and 14 patients with normal X-ray findings but abnormal chest CT findings. (**C**) OS of patients with newly diagnosed acute leukemia according to both abnormal chest CT and chest X-ray findings, abnormal chest CT findings and normal chest X-ray findings, and both normal chest CT and chest X-ray findings.

Next, we explored the association between baseline chest X-ray abnormalities and OS. Patients with abnormal baseline chest X-ray findings (*n* = 33), had significantly shorter OS than those with normal baseline chest X-ray findings (*n* = 17) (median OS, 8.7 months vs. not reached; *p* = 0.006) (Figure 2A). On multivariate analysis, the adverse survival impact of an abnormal baseline chest X-ray was retained (*p* = 0.005) when accounting for white blood cell count, *TP53* mutation status, ECOG performance status, acute leukemia subtype (i.e., AML vs. ALL), age ≥ 60, current infection, and co-morbidities, all of which are risk factors in acute leukemia (Supplemental Table S6A) [19,20].

We then assessed whether considering both baseline chest CT findings and chest X-ray findings affected the association between abnormal baseline chest X-ray findings and OS. Because only three patients had both normal chest X-ray and chest CT findings, we simplified the model by comparing patients with both abnormal chest X-ray and abnormal CT findings with all other patients. On multivariate analysis, patients with both abnormal chest X-ray and chest CT findings had worse overall survival than patients with normal chest X-ray findings but either normal or abnormal chest CT findings (*p* = 0.005) (Supplemental Table S6B) (Figure 2B). Because all patients with abnormal chest X-ray findings also had abnormal chest CT findings, this approach resulted in patient groupings that were identical to those generated by the abnormal vs. normal chest X-rays variable alone.

We further compared OS between patients who had both abnormal chest X-ray and chest CT findings (*n* = 33), patients with normal chest X-ray findings but abnormal chest CT findings (*n* = 14), and patients with both normal chest X-ray and chest CT findings (*n* = 3). Patients with abnormalities on both imaging studies had inferior OS (median OS, 8.37 months vs. not reached vs. not reached; *p* = 0.022) (Figure 2C). The causes of death are listed in Supplemental Table S7.

## 4. Discussion

Our study demonstrated that patients with newly diagnosed acute leukemia often had abnormal baseline chest imaging. Chest CT demonstrated superior sensitivity, detecting abnormalities in 94% of patients compared to 66% by chest X-ray. Moreover, most patients (*n* = 30, 60%) in this analysis had normal or minimal chest X-ray findings compared to their chest CT findings. The presence or absence of imaging abnormalities was not significantly associated with clinical symptoms or laboratory data.

Importantly, all clinically significant CT-detected abnormalities prompted additional interventions, including specialty consultations, procedures, treatment modifications, or closer monitoring. These findings suggest that incorporating readily available, non-invasive baseline chest CT imaging may provide a more comprehensive assessment and could potentially improve clinical management for patients with acute leukemia. Our study highlights this diagnostic gap, potentially supporting prospective studies to determine the potential for routine chest CT evaluation for patients with newly diagnosed acute leukemia.

Patients with acute leukemia are susceptible to both infectious and non-infectious pulmonary manifestations. Readily available chest CT findings suggestive of infection could prompt earlier treatment decisions and clinical interventions, reducing potentially preventable complications. In our cohort, 16 of 47 (34%) patients had chest CT abnormalities without fever or microbiological evidence of obvious infection, but 33 patients had opacities and 14 had nodules and/or effusions. Indeterminate small nodules (28%) and more prominent nodules (16%) were observed on chest CT [21]. About a quarter of patients (14 of 50) underwent BAL, with four having findings confirming infection. These results also underscore the potential value of incorporating flow cytometry and cytology assessment when bronchoscopy or BAL can be safely performed to enhance accuracy in distinguishing infection from leukemic infiltration.

In this exploratory analysis intended to generate hypotheses for future research, chest imaging abnormalities were significantly associated with OS. Patients with chest X-ray abnormalities had worse survival compared to patients with normal chest X-rays, and chest CT abnormalities further stratified survival among patients with normal or abnormal chest X-ray findings. Patients with both chest X-ray and chest CT abnormalities had the poorest outcomes. The adverse survival impact of imaging abnormalities in patients with acute leukemia was maintained on multivariate analysis. However, this hypothesis-generating observation requires prospective validation. Because patients with abnormal imaging findings likely represented a clinically more vulnerable population, the observed associations may reflect underlying disease severity, with imaging abnormalities serving as surrogate markers of illness severity rather than independent prognostic factors. Furthermore, only three patients had both normal CT and normal chest X-ray.

Our study has important limitations inherent to retrospective analyses. Our inclusion criteria were strict, restricting imaging to a narrow time frame before starting induction therapy and requiring that patients underwent both chest X-ray and chest CT; thus, our sample size is small. Patients who underwent chest X-ray and/or chest CT within a wider time frame were not included. Additionally, we included only patients with newly diagnosed acute leukemia, excluding patients with relapsed or refractory disease, who might have a higher prevalence of infection or other pulmonary abnormalities. However, data were obtained directly from the electronic medical record, so loss of patient data was minimal. Our data highlight that despite normal or minimal chest X-ray findings, chest CT identified actionable pulmonary pathology in more than half of our cohort. However, retrospective “actionability” determination is a limitation.

An important limitation is that we could not control for potential clinician-dependent practice regarding the decision to obtain baseline chest CT imaging. We found that 66% of patients had baseline chest radiograph abnormalities and 62% experienced fever during the week preceding induction therapy, which is consistent with similar rates reported in prior studies of acute leukemia [22,23,24,25,26]. Patsios et al. reported abnormal baseline chest radiographs in 31 of 40 (77.5%) patients with acute leukemia [22]. Compared with a reported pleural effusion rate of 47.5% in a study by Patsios et al. [22], pleural or pericardial effusions were identified on chest CT in 44% of patients in our cohort. Similarly, fever is among the most common manifestations of acute leukemia [23,24,25] and has been reported in approximately 80–90% of patients with acute leukemia [22,24,26]. Our prespecified eligibility criterion of undergoing both a chest CT and chest X-ray within four days before beginning induction chemotherapy provided us with a unique cohort of 50 patients among the 1190 screened patients. Thus, although we cannot exclude the possibility of clinician-dependent practice, retrospective patient inclusion was determined solely by objective database criteria.

All chest CT examinations were performed without intravenous contrast, which is considered appropriate for the initial evaluation of immunocompromised patients with acute respiratory illness according to The American College of Radiology Appropriateness Criteria [27]. Contrast-enhanced CT may provide additional diagnostic information in selected clinical settings [27], and this would require specialized procedures. For example, CT pulmonary angiography can identify radiographic signs of angioinvasion, including the vessel occlusion sign, which may support the diagnosis of invasive pulmonary fungal disease [28].

In current clinical practice, a non-contrast chest CT is the standard initial imaging protocol for general pulmonary assessments including evaluation of fungal pulmonary infections [28], because pulmonary inflammation, edema, and other parenchymal abnormalities are generally well visualized without intravenous contrast [29].

Radiographic abnormalities in acute leukemia patients can be nonspecific, but earlier detection of an active process can change management. Our study supports prospective studies to determine whether pretreatment chest CT could become standard of care for baseline assessment in this population. Although leukemic or other non-infectious manifestations may be found, the major impetus would be to help identify occult infections or findings that could worsen after treatment.

This study reflects the challenge of balancing early detection of occult fungal infection against the risks, costs, and potential treatment delays associated with acting on non-contrast chest CT findings without microbiologic confirmation by BAL. In accordance with NCCN guidelines [18] and institutional practice, all patients received antibacterial, antifungal, and antiviral prophylaxis throughout induction and consolidation therapy cycles; however, the use of baseline CT scans may increase antimicrobial exposure with escalation of antibiotic regimens. Prospective studies are warranted to evaluate the cost effectiveness and impact on mortality of this practice. Large prospective registry studies are needed to improve differentiation between pulmonary infection and noninfectious conditions, such as leukemic lung infiltration and treatment-related pulmonary toxicity, which can present with overlapping clinical and radiographic features. Importantly, all imaging studies in the present cohort were obtained before beginning leukemia-directed therapy, eliminating drug-related pulmonary toxicity as a source of diagnostic uncertainty. While leukemic lung infiltration may mimic opportunistic pulmonary infection, ongoing efforts are focused on developing noninvasive diagnostic approaches to reduce the need for invasive procedures used to confirm invasive mold infections, including the evaluation of exhaled volatile organic compounds [30].

Larger prospective systematic studies of pre-induction thoracic imaging in this population would be helpful in the future.

## 5. Conclusions

Performing chest CT before initiating chemotherapy may provide clinically meaningful information beyond the information obtained from chest X-ray, possibly influencing changes in patient management. Our findings may support further investigation into whether chest CT could serve as an alternative to routine pre-chemotherapy chest X-ray in newly diagnosed acute leukemia.

## Figures and Tables

**Table 1:** Baseline characteristics.

Characteristic	ALL (*N* = 11)	AML (*N* = 39)	Total (*N* = 50)
Age, years; median (range)	55 (18–82)	68 (20–94)	68 (18–94)
Male, *n* (%)	8 (73)	20 (51)	28 (56)
Imaging findings ^‡^			
Abnormal chest X-ray, *n* (%)	6 (55)	27 (69)	33 (66)
Abnormal chest CT, *n* (%)	11 (22)	36 (72)	47 (94)
Abnormal chest X-ray and chest CT	6 (55)	27 (69)	33 (66)
Normal chest X-ray, but abnormal chest CT	5 (45)	9 (23)	14 (28)
Both chest X-ray and chest CT normal	0 (0)	3 (8)	3 (6)
Fever present at baseline, *n* (%) ^†^	4 (36)	15 (38)	19 (38)
White blood cell count (K/uL), median (range)	3.5 (1.5–45.5)	7 (0.7–141.0)	5.7 (0.7–141.0)
Hemoglobin (g/dL), median (range)	9.3 (8.0–14.1)	8.6 (6.6–13.3)	8.9 (6.6–14.1)
Platelets (K/uL), median (range)	35 (13–88)	52 (7–409)	49 (7–409)
Absolute neutrophil count (K/uL), median (range)	0.9 (0.1–2.8)	0.9 (0–35.5)	0.9 (0–35.5)
Lactate dehydrogenase (U/L), median (range)	504 (158–4590)	531 (220–6776)	518 (158–6776)
Peripheral blood blasts (%), median (range)	8 (0–85)	19 (0–90)	19 (0–90)
Bone marrow blasts (%); median (range)	78 (3–89)	55 (5–91)	65 (3–91)
Cytogenetics, *n* (%)			
Diploid	0 (0)	12 (31)	12 (24)
Core-binding factor	0 (0)	3 (8)	3 (6)
−5/5q or −7/7q	0 (0)	12 (31)	12 (24)
Hyperdiploid	6 (55)	0 (0)	6 (12)
Ph+	4 (36)	0 (0)	4 (8)
Hypodiploid	1 (9)	0 (0)	1 (2)
Other	0 (0)	9 (23)	9 (18)
Unknown	0 (0)	3 (8)	3 (6)
Gene mutations *			
*TP53*	1 (14)	10 (29)	11 (26)
*U2AF1*	0 (0)	1 (5)	1 (4)
*TET2*	4 (44)	17 (52)	21 (50)
*ASXL1*	6 (60)	6 (20)	12 (30)
*DNMT3A*	0 (0)	9 (27)	9 (23)
*FLT3*-ITD	0 (0)	5 (14)	5 (11)
*FLT3*-D835	0 (0)	2 (6)	2 (5)
*IDH1*	0 (0)	5 (15)	5 (13)
*IDH2*	1 (14)	6 (15)	7 (17)
*JAK2*	1 (9)	5 (15)	6 (15)
*NPM1*	0 (0)	6 (18)	6 (15)
*PTPN11*	1 (13)	2 (7)	3 (8)
*IKZF1*	1 (20)	0 (0)	1 (9)

Abbreviations: ALL—acute lymphoblastic leukemia; AML—acute myeloid leukemia; CT—computed tomography. * Percentages calculated from non-missing observations. ^‡^ All patients with abnormal chest X-ray findings also had abnormal chest CT findings. ^†^ Fever was defined as temperature ≥ 100.4 °F.

**Table 2:** Chest imaging abnormalities.

**(** **A) Proportions of Abnormalities Identified by Chest X-ray and Chest CT**				
Imaging abnormality *	**Chest X-ray, *n* (%)**	**Chest CT, *n* (%)**		***p*** **-value**
Any abnormality	33 (66)	47 (94)		<0.001
Pleural or pericardial effusions	13 (26)	22 (44)		0.059
Nodules	6 (12)	19 (38)		0.003
Opacities	27 (54)	33 (66)		0.220
Cavitations	1 (2)	2 (4)		0.564
**(B) Reason Chest CT was Ordered (*N* = 50)**				
**Reason**	**Chest X-ray Normal (*N* = 17)**		**Chest X-ray Abnormal (*N* = 33)**	
Cough	3		1	
Chest X-ray abnormal ± fevers/cough	0		26	
Fever ± cough/dyspnea	12		5	
Hemoptysis	0		1	
Baseline assessment before chemotherapy	2		0	

* Chest X-ray abnormalities included cavitations, nodules, opacities, infiltrates, and effusions.

**Table 3:** Concordance and comparisons between chest X-ray and chest CT findings (*N* = 50).

			% Agreement	Kappa *p*-Value	McNemar’s *p*-Value
**Chest CT**	**Chest X-Ray**				
	**No**	**Yes**			
**Opacities**					
No	10	7	60%	0.096	0.180
Yes	13	20			
**Cavitation**					
No	47	1	94%	0.582	0.564
Yes	2	0			
**Effusions (Pleural and/or Pericardial)**					
No	24	4	66%	0.017	0.029
Yes	13	9			
**Nodules**					
No	30	1	70%	0.007	0.001
Yes	14	5			

**Table 4:** Imaging findings and the impact on clinical care.

**Normal Chest X-ray with Abnormal Chest CT**	14
Imaging description for actionable chest CT findings	10
Effusion, opacity	2
Pleural effusion, opacity, lung nodule	2
Pleural effusion, opacity, septal thickening	1
Opacity	4
Opacity, small lung nodule	1
Action taken	10
Antibiotic regimen change	4
Active surveillance	1
Both infectious disease team consult and antibiotics change	1
Both antibiotics change and active surveillance	4
Imaging description was non-specific, non-actionable for clinically insignificant CT findings	4
Atelectasis	1
Tiny/nonspecific indeterminate nodules	2
Granuloma	1
**Both Abnormal Chest X-ray and Abnormal Chest CT**	33
**Both Abnormal Chest X-ray and Abnormal Chest CT with Minimal Chest X-ray findings relative to Chest CT ^†^**	16
Imaging description for actionable chest CT findings	16
Pleural effusion	1
Pleural effusion, enlarged lymph node	1
Pleural effusion, lung nodule, enlarged lymph node	1
Pleural effusion, opacity	3
Pleural effusion, opacity, lung nodule	1
Pleural effusion, opacity, cavitary lesion	1
Pleural effusion, opacity, enlarged lymph node	1
Opacity	4
Opacity, enlarged lymph node	2
Opacity, lung nodule	1
Action taken	16
Infectious disease team consult, antibiotic regimen change	7
Infectious disease team consult, antibiotic regimen change, planned bronchoscopy, active surveillance	1
Antibiotic regimen change, active surveillance	2
Antibiotic regimen change	6
**Both Chest X-Ray ^‡^ and Chest CT abnormal but Chest X-ray is not minimal relative to chest CT**	**17**
**Normal chest X-ray and chest CT**	**3**

**^†^** Chest X-ray findings were considered minimal relative to chest CT findings when chest X-ray findings were normal, indeterminate, or demonstrated substantially less extensive disease than that identified on chest CT. ^‡^ Chest X-ray findings were comparable to chest CT findings. Chest CT provided no additional clinical information and did not alter clinical decision-making.

## Data Availability

The data presented in this study are available on request from the corresponding author. The authors will provide access to deidentified participant-level data underlying this article to researchers who submit an academically valid proposal to interpret, verify, or extend this research, provided that such access does not violate privacy, data encumbrance, intellectual property, or other legal, regulatory, or contractual confidentiality obligations. Data sharing will be subject to a data use agreement with The University of Texas MD Anderson Cancer Center.

## Supplementary Materials

The following supporting information can be downloaded at: https://www.mdpi.com/article/10.3390/cancers18193155/s1, Table S1: Treatment regimens of the 39 AML and 11 ALL patients; Table S2: Clinical Correlation with Imaging; Table S3: Clinical and imaging findings of the 14 patients who underwent bronchoscopy or bronchoalveolar lavage; Table S4: Rate of agreement on concern for infection and actionability; Table S5: Clinical laboratory values associated with imaging abnormalities; Table S6: Multivariate analyses of survival according to baseline imaging features; Table S7: Causes of Death (*n* = 29); Figure S1: Patients with Single and Multiple Abnormalities on Chest Imaging among (A): patients with an abnormal chest X-ray, (B): an abnormal chest CT, and (C): a normal chest X-ray but abnormal chest CT.
