# Supplementary material for: The Utility of Computed Tomography of the Chest at the Time of Induction Chemotherapy in Patients with Acute Leukemia
Source: Cancers (Basel). 2026 Sep 29;18(19):3155. doi: 10.3390/cancers18193155 (PMC13664304; doi:10.3390/cancers18193155)
Supplement: Supplementary file 1 [file cancers-18-03155-s001.zip › cancers-4414224-supplementary.pdf]

Supplemental Materials

Supplemental Table S1. Treatment regimens of the 39 AML and 11 ALL patients.

Regimen	Number
AZA-enasidenib	1
AZA-sorafenib	1
AZA-quizartinib	1
Blinatumomab-ponatinib	2
CLAD-LDAC	2
CLAD-LDAC-VEN	2
CLIA	4
CLIA-sorafenib	1
DEC-VEN-gilteritinib	1
DEC-R	2
EPOCH-R	1
FLAG	1
FLAG-GO	1
FLAG-Ida	1
FLAG-Ida-GO	1
FLAG-IDA-VEN	1
HyperCVAD-blinatumomab-ofatumomab	2
HyperCVAD	1
HyperCVAD-blinatumomab	1
HyperCVAD-dasatinib	1
IA	1
IA-nivolumab	2
LDAC-BP1001	1
MiniHVD-Inotuzomab	1
R-hyperCVAD	1
R-hyperCVAD-dasatinib	1
SGI-110-CLAD	1
SGI-110-Ida	1
VEN-DEC	11
VEN-AZA	2

Abbreviations: **AZA** = azacitidine; **VEN** = venetoclax; **DEC** = decitabine; **CLAD** = cladribine; **LDAC** = low-dose cytarabine; **CLIA** = cladribine, idarubicin, cytarabine; **IA** = idarubicin, cytarabine; **FLAG** = fludarabine, cytarabine, G-CSF; **FLAG-Ida** = FLAG plus

idarubicin; **GO** = gemtuzumab ozogamicin; **HyperCVAD** = hyperfractionated cyclophosphamide, vincristine, doxorubicin, dexamethasone; **EPOCH-R** = etoposide, prednisone, vincristine, cyclophosphamide, doxorubicin plus rituximab; **MiniHVD** = reduced-intensity hyperfractionated cyclophosphamide, vincristine, dexamethasone; **Inotuzomab** = inotuzumab ozogamicin; **Blinatumomab** = CD19-directed BiTE antibody; **Ponatinib**, **Dasatinib**, **Sorafenib**, **Quizartinib**, **Gilteritinib** = tyrosine kinase inhibitors; **Ida** = idarubicin; **SGI-110** = guadecitabine; **R** = rituximab; **BP1001** = liposomal Grb2 antisense oligonucleotide.

Supplemental Table S2. Clinical Correlation with Imaging.

	CT Chest and Chest X-ray Abnormalities						
	Both Abnormal (N = 33)		CT Chest Abnormal and Chest X-ray Normal (N = 14)		Both Normal (N=3)		
**Characteristic†**	**N**	**%**	**N**	**%**	**N**	**%**	**p-value**
Fever within 1-week							0.263
No	15	45	4	29	0	0	
Yes	18	55	10	71	3	100	
On oxygen							0.065
No	15	45	10	71	3	100	
Yes	18	55	4	29	0	0	
Cough							0.764
No	24	73	10	71	3	100	
Yes	9	27	4	29	0	0	
Bronchoscopy or BAL done							0.769
No	23	70	10	71	3	100	
Yes	10	30	4	29	0	0	

Abbreviations: BAL, bronchoalveolar lavage

†Five patients had a fever, cough and oxygen requirement

Supplemental Table S3. Clinical and imaging findings of the 14 patients who underwent bronchoscopy or bronchoalveolar lavage.

Patient	Confirmed infection?	Cough	Oxygen	Chest X-Ray findings	CT chest findings
1	Mycobacterium intracellulare	Yes	Yes	Opacity	Opacity
2	MRSA and Pseudomonas	No	Yes‡	Bilateral opacities in the lungs, left greater than right concerning for areas of pneumonia. Small left pleural effusion.	Mild bilateral patchy ground glass opacities. Irregular small pulmonary nodules in bilateral upper lobes with internal cystic changes.
3	Pseudomonas	Yes	Yes	Moderate right-sided pleural effusion.	Opacity
4	Rhinovirus enterovirus	Yes	No	Negative	Bilateral ground glass opacities.
5†	No	No	Yes	Small nodular opacities in the left lung and small pleural effusions	Multiple bilateral pulmonary nodules. The largest nodules have ground glass halos. Small bilateral pleural effusions. Mild pulmonary edema.
6†	No	No	Yes	Pulmonary opacities	Focal consolidation in the middle lobe and more diffuse ground glass

					opacities in the right lower. Nonspecific pulmonary nodules in the right lung up to 3 mm in diameter.
7†	No	Yes	Yes	Negative	Areas of clustered nodularity within lungs. Subpleural consolidative opacity at the right base.
8†	No	No	Yes	Negative	Subtle diffuse ground glass opacities and minimal interlobular septal thickening. Small left pleural effusion.
9	No	No	No	Negative	Focal consolidation and tethering in the right lower lobe associated with pleural thickening and nodularity and small effusion. Small lymph nodes.
10	No	Yes	Yes	Bilateral lung opacities, small bilateral effusions	Bilateral lung opacities may represent infection or pulmonary edema.
11	No	No	Yes‡	Multifocal opacity in the right lung	Right greater than left patchy and nodular pulmonary opacities suspicious for infection
12	No	No	Yes	Bilateral lung radiopacities	Consolidative opacities in the lower lobes. Diffuse

					ground glass opacities in the lungs.
13	No	Yes	No	Poorly marginated opacities in the right lung adjacent to the right hemidiaphragm.	Consolidative opacities in the right middle lobe with ground glass opacities in the right upper lobe. Small right pleural effusion.
14	No	No	No	Irregular opacity overlying the left hemidiaphragm	Small left pleural effusion

†Patients #6, #7, #8, #9 had suspected fungal lung infections based on imaging findings, despite all 4 having negative fungal cultures, and negative aspergillus antigen on BAL and blood.
‡No fever initially, but developed a fever many days later

Supplemental Table S4. Rate of agreement on concern for infection and actionability.

	Rater 1 vs. Rater 2 vs. Rater 3* (n = 3)		Rater 1 vs. Rater 2 (n = 2)	
	Kappa	p-value	Kappa	p-value
Are CT Findings Concerning for Infection?	0.9316	<0.001	0.9467	< 0.001
Are CT Findings Actionable?	0.9316	0.001	0.9467	0.001

*Third doctor only had 1 observation

Supplemental Table S5. Clinical laboratory values associated with imaging abnormalities.

Characteristic	Positive X-ray and CT chest (n=33)	Abnormal CT Chest and normal X-ray (n=14)	Both negative (n=3)	p-value
White blood cell count				0.308
Mean (SD)	17.0 (30.7)	26.6 (33.3)	16.6 (24.2)	
Median (range)	4.3 (0.7-141.0)	18.6 (1.5-126.0)	3.5 (1.8-44.6)	
Hemoglobin				0.508
Mean (SD)	9.0 (1.6)	9.5 (1.6)	8.6 (2.0)	
Median (range)	8.6 (6.6-14.1)	9.1 (7.0-12.2)	8.6 (6.7-10.6)	
Platelet count				0.794
Mean (SD)	62.7 (72.4)	82.9 (91.2)	58.7 (22.3)	
Median (range)	48.0 (7.0-409.0)	47.0 (13.0-297.0)	70.0 (33.0-73.0)	
Neutrophils (%)				0.086
Mean (SD)	28.9 (25.2)	15.0 (15.6)	15.0 (20.2)	
Median (range)	21.0 (1.0-79.5)	7.5 (0.0-45.0)	7.0 (0.0-38.0)	
Absolute neutrophil count				0.161
Mean (SD)	2.7 (6.2)	2.4 (3.4)	0.3 (0.3)	
Median (range)	1.1 (0.0-35.5)	0.8 (0.0-10.6)	0.2 (0.0-0.7)	
Serum lactate hydrogenase				**0.021**
Mean (SD)	754.1 (1166.7)	1458.3 (1266.1)	612.0 (315.3)	
Median (range)	396.0 (158.0-6776.0)	1007.0 (324.0-4590.0)	759.0 (250.0-827.0)	
Blasts, peripheral blood (%)				0.204
Mean (SD)	24.9 (27.8)	38.0 (25.6)	39.3 (45.1)	
Median (range)	14.0 (0.0-90.0)	37.5 (2.0-85.0)	28.0 (1.0-89.0)	
Blasts, bone marrow (%)				0.233
Mean (SD)	53.2 (24.3)	66.6 (21.9)	49.7 (36.0)	
Median (range)	55.0 (5.0-89.0)	72.0 (3.0-91.0)	33.0 (25.0-91.0)	
Monocytes (%)				0.547
Mean (SD)	10.0 (13.2)	16.2 (17.0)	5.0 (7.0)	
Median (range)	3.6 (0.0-43.0)	11.5 (0.0-55.0)	2.0 (0.0-13.0)	
PTT				0.160
Mean (SD)	36.0 (7.5)	34.1 (8.6)	27.1 (6.0)	
Median (range)	35.2 (22.4-55.6)	32.5 (22.5-50.0)	23.9 (23.3-34.0)	
Serum albumin*				0.796
Mean (SD)	3.7 (0.6)	3.7 (0.6)	3.9 (0.2)	
Median (range)	3.7 (2.3-4.8)	3.7 (2.9-4.7)	3.9 (3.8-4.1)	
Alkalase phosphatase				0.870
Mean (SD)	107.8 (94.4)	95.6 (36.9)	89.7 (34.7)	
Median (range)	75.0 (49.0-520.0)	87.0 (45.0-174.0)	94.0 (53.0-122.0)	
INR				0.556
Mean (SD)	1.2 (0.2)	1.3 (0.3)	1.1 (0.1)	
Median (range)	1.2 (0.9-2.0)	1.2 (1.0-1.8)	1.2 (1.0-1.2)	

*Five patients with a positive X-ray and CT chest, and one patient with an abnormal CT Chest and normal X-ray had missing albumin values

Supplemental Table S6. Multivariate analyses of survival according to baseline imaging features.

**A. Model 1: Baseline Chest X-ray Features**	**HR (95% CI)**	**p value**
Abnormal X-ray vs. normal X-ray*	3.67 (1.33-10.09)	0.005
White Blood Cell Count	1.00 (0.99-1.02)	0.667
Age > 60 years vs. Age ≤ 60 years	0.26 (0.08-0.87)	0.029
TP53 mutation status	0.53 (0.15-1.93)	0.339
ECOG performance status (0-2 vs ≥3)	9.94 (1.70-58.2)	0.011
Acute leukemia subtype (AML vs ALL)	5.60 (1.05-29.92)	0.044
Renal co-morbidities	3.10 (0.14-68.91)	0.475
Cardiac co-morbidities	3.40 (1.28-8.93)	0.014
Pulmonary co-morbidities	0.59 (0.23-1.51)	0.268
Neurological comorbidities	10.86 (2.67-44.12)	0.001
Metabolic comorbidities	0.42 (0.14-1.26)	0.123
Current infection†	0.72 (0.21-2.45)	0.210
**B. Model 2: Baseline Chest CT and X-ray Features**		
Abnormal Chest CT and abnormal Chest X-ray vs not*	3.67 (1.33-10.09)	0.005
White Blood Cell Count	1.00 (0.99-1.02)	0.667
Age > 60 years vs. Age ≤ 60 years	0.26 (0.08-0.87)	0.029
TP53 mutation status	0.53 (0.15-1.93)	0.339
ECOG performance status (0-2 vs ≥3)	9.94 (1.70-58.2)	0.011
Acute leukemia subtype (AML vs ALL)	5.60 (1.05-29.92)	0.044
Renal co-morbidities	3.10 (0.14-68.91)	0.475
Cardiac co-morbidities	3.40 (1.28-8.93)	0.014
Pulmonary co-morbidities	0.59 (0.23-1.51)	0.268
Neurological comorbidities	10.86 (2.67-44.12)	0.001
Metabolic comorbidities	0.42 (0.14-1.26)	0.123
Current infection†	0.72 (0.21-2.45)	0.210

*Of the 50 patients, 33 patients had an abnormal chest X-ray and 47 had an abnormal chest CT. Because all patients with an abnormal chest X-ray also had an abnormal chest CT, the categories generated by the variable "abnormal X-ray vs normal chest X-ray" in Model 1 were identical to those generated by the variable "Abnormal Chest CT and a abnormal chest X-ray" in Model 2, resulting in identical patient groupings.
†Current infections included pneumonia (n=5), fever or unknown origin (n=2), and cellulits/abscess (n=1)

Current infection0.72 (0.21-2.45)0.210

Supplemental Table S7. Causes of Death (*n* = 29).

**Cause of Death Category**	**Number of Deaths**	**Representative Causes**
Infection +/- sepsis	n=9	
Multiorgan failure (MOF)	n=5	MOF associated with infection, respiratory failure, or systemic illness
Hospice/comfort-focused care	n=5	Transition to hospice and discharge for end-of-life care
Acute leukemia progression	n=2	
Unknown	n=8	

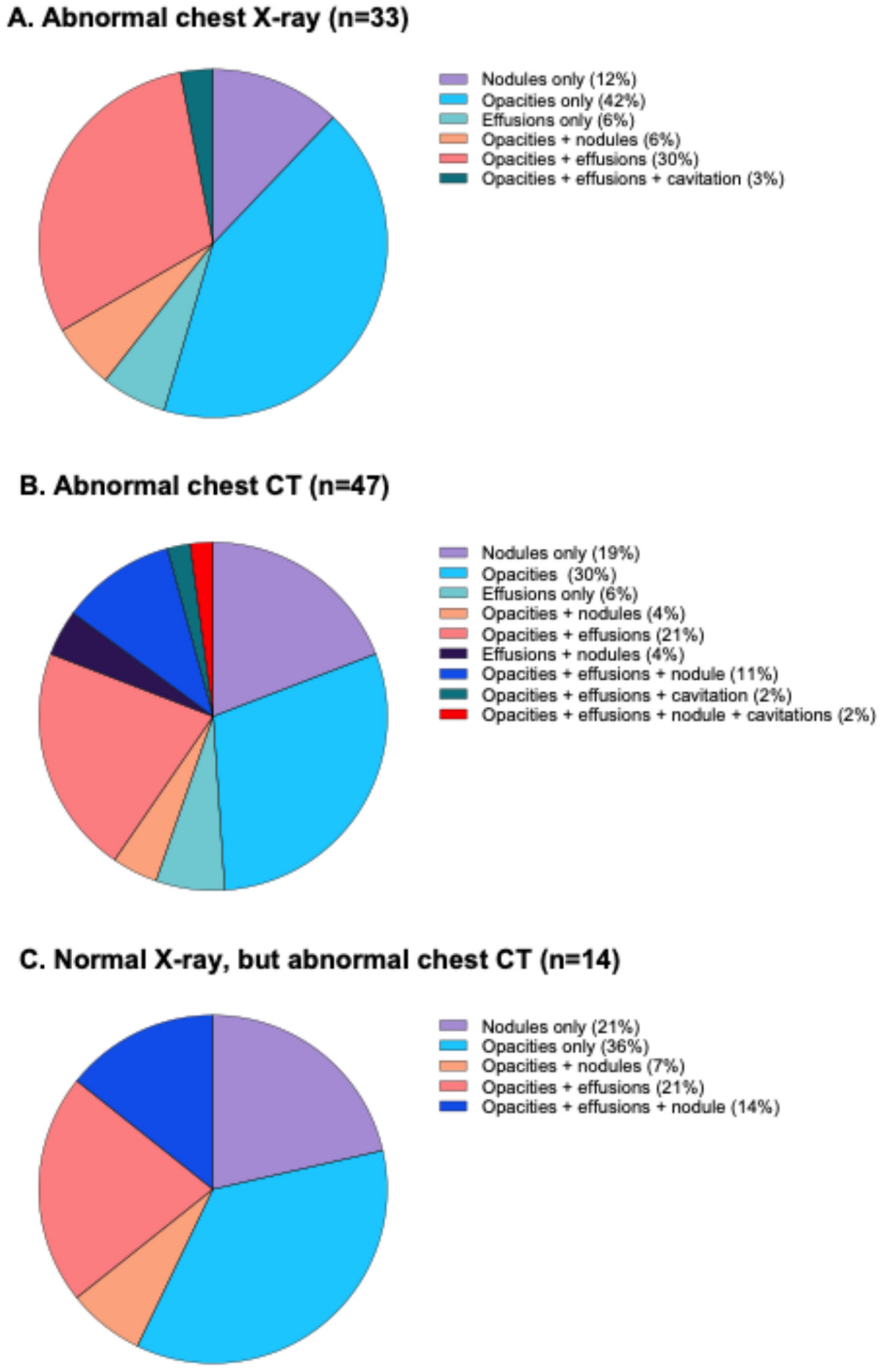

Supplemental Figure S1. Patients with Single and Multiple Abnormalities on Chest Imaging among (A): patients with an abnormal chest X-ray, (B): an abnormal chest CT, and (C): a normal chest X-ray but abnormal chest CT.
